# Effects of a Combination of Thyme and Oregano Essential Oils on TNBS-Induced Colitis in Mice

**DOI:** 10.1155/2007/23296

**Published:** 2007-10-10

**Authors:** Alexandra Bukovská, Štefan Čikoš, Štefan Juhás, Gabriela Il’ková, Pavol Rehák, Juraj Koppel

**Affiliations:** Institute of Animal Physiology, Slovak Academy of Sciences, Šoltésovej 4-6, Košice 04001, Slovakia

## Abstract

We examined the anti-inflammatory effects of the combination of thyme and oregano essential oil dietary administered at three concentrations (0.4% thyme and 0.2% oregano oils; 0.2% thyme and 0.1% oregano oils; 0.1% thyme and 0.05% oregano oils) on mice with TNBS-induced colitis. Treatment of colitic animals with the essential oils decreased the mRNA levels of pro-inflammatory cytokines IL-1β, IL-6, GM-CSF, and TNFα, especially after application of the medium dose. The medium dose of the essential oils significantly lowered the amount of IL-1β and IL-6 proteins too. Moreover, administration of the medium dose decreased the mortality rate, accelerated the body weight gain recovery, and reduced the macroscopic damage of the colonic tissue. Our results indicate that combined treatment with appropriate concentrations of thyme and oregano essential oils can reduce the production of proinflammatory cytokines, and thereby attenuate TNBS-induced colitis in mice.

## 1. INTRODUCTION

Intestinal inflammatory diseases are a serious problem in human as well as veterinary medicine. The etiology of these diseases is often multifactorial and the underlying molecular mechanisms are poorly understood [1]. The current medicinal therapies for inflammatory gut diseases involve treatment with nonsteroidal anti-inflammatory drugs, antibiotics, corticosteroids, and immunosuppressant, but the application of these drugs is limited due to their toxicity and side effects [2]. Therefore, there is an increased interest in finding an alternative treatment with fewer side effects.

There is evidence supporting the therapeutic usefulness of oral administration of various plant extracts in inflammatory diseases of the gut. Experimental data obtained in mouse and rat models of colitis suggest that the beneficial effects of the plant extracts could be mediated by their effects on mucosal cytokines production or/and action [3–9]. Increased levels of proinflammatory cytokines [IL-1, IL-6, IL-8, TNF*α*, IL-12, and IFN*γ*] were found in inflamed intestinal mucosa in various animal models and humans [10–13], as well as in farm animals [14, 15].

Thyme (*Thymus vulgaris* L.) and oregano (*Origanum vulgare* L.) are aromatic plants of the Mediterranean flora commonly used as spices and for medicinal purposes. Like other various Thymus species, thyme is traditionally used for its antiseptic, antispasmodic, and antitussive effects. Furthermore, thyme possesses antimicrobial, antifungal, antioxidative, and antiviral properties [16–19]. The essential oil derived from thyme (*T. vulgaris* L.) is a mixture of monoterpenes and one of the main compounds of this oil is a natural terpenoid thymol [20]. Thymol exhibits multiple biological activities including anti-inflammatory [21], immunomodulating [22], antioxidant [23], antibacterial [24, 25], antifungal [26], and free radical scavenging properties [27]. Oregano is recognized for its potential therapeutic role because of its diaphoretic, carminative, antispasmodic, antiseptic, and tonic properties. Oregano, (*Origanum syriacum* L.) similar to thyme, evinces antioxidant and antimicrobial activities [28] and some reports deal with its antimutagenic and anticarcinogenic effects [29]. Origanum essential oil is obtained by steam distillation of *O. vulgare* and its major compounds are carvacrol and thymol [30]. Origanum essential oil is known to possess antimicrobial, antifungal, and antioxidant activities [31, 32].

The aim of our study was to examine possible beneficial effects of thyme and oregano essential oils on intestinal inflammation. The results of our preliminary experiment suggested that the administration of thyme oil in combination with oregano oil could be more effective in improvement of trinitrobenzene sulphonic acid (TNBS)-induced colitis than the separate administration of these essential oils. In the present study, we evaluated further the effect of administration of three different doses of thyme and oregano oil combination on TNBS-induced colitis in mice.

## 2. MATERIALS AND METHODS

### 2.1. Animals and treatment

Male 7-week-old Balb/c mice weighing 18–25 g were purchased from Velaz (Prague, Czech Republic). The animals were maintained under standard conditions of temperature $\left(21\pm 1^{{}^{\circ}}\text{C}\right)$, relative humidity (55±10%), and 12 hours/12 hours light/dark cycle. All mice were housed in specific pathogen-free conditions. All animal experimentations were reviewed and approved by the Ethical Committee of the Institute of Animal Physiology.

After a period of adaptation, weight-matched animals were randomized into five groups: group A (20.2±0.971 g), group B (20.6±0.75 g), and group C (20.71±0.844 g), mice with TNBS-induced colitis treated with three different doses (see Table 1) of thyme and oregano oil combination; group D (21.03±1.025 g), mice with TNBS-induced colitis; group E (18.74±1.189 g), sham-treated mice.

Thyme aromatic oil (*Thymi aetheroleum*-Ph.Eur. 4) and oregano aromatic oil (*Origani aetheroleum*) were purchased from Calendula, (Nová L'ubovňa, Slovakia; thyme aromatic oil: lot 5-015-003-10-04; oregano aromatic oil: lot 5-027-007-10-04). The thyme aromatic oil contained about 48% of. p-cymene and 24% of thymol, and the oregano aromatic oil contained about 55% of carvacrol. 2,4,6-trinitrobenzene sulphonic acid (TNBS) was purchased from Fluka Chemie (Buchs, Switzerland). The thyme oil and oregano oil were mixed with the diet at concentrations as shown in Table 1. Both aromatic oils were suspended in edible soya oil (Brölio, Hamm, Germany) and added to powdery commercial rodent diet (diet for laboratory mice and rats SPF, M1; Frantisek Machal, Ricmanice, Czech Republic). In the TNBS and sham groups, edible soya oil was mixed with the powdery rodent diet at a concentration of 1% (wt/wt). Diets were fed *ad libitum* throughout the experiment, starting 6 days before administration of TNBS.

### 2.2. Induction of colitis, sample preparation, macroscopical and histological assessment

The mice were anesthetized with ketamine and xylazine, and colitis was induced by intrarectal administration of 120 mg/kg of the hapten reagent TNBS (Fluka Chemie) in 50% ethanol, and they were then kept in a vertical position for 30 seconds. The sham group received 50% ethanol alone using the same technique. The total injection volume was 30 *μ*L. Development of colitis was assessed daily by measurement of body weight. The mortality rate was observed during this study. The mice were killed by cervical dislocation 7 days after TNBS administration. The colons were removed, cut longitudinally, and cleared of fecal material with gentle spray of 0.9% saline solution. The extent of mucosal damage was assessed using the colon macroscopic scoring system adapted from Wallace et al. [33]. *Ulceration* : (1) focal hyperemia, no ulcer; (2) ulceration, no hyperemia/bowel wall thickening; (3) ulceration, inflammation at one site; (4) ulceration, inflammation at 2 or more sites; (5) major injury *>* 1 cm; 6–10 major damage *>* 2 cm. *Adhesion*: (1) minor (colon easily separated from other tissue); (2) major. *Diarrhea*: (1); *Bowel wall thickening*: (1). Representative samples from each experimental group were histologically evaluated. Colon tissues were fixed in 4% formalin in 0.1 M phosphate buffer, dehydrated with increasing concentrations of ethanol, embedded in paraffin, and sectioned. Sections (4–6 *μ*m thick) were mounted on slides, cleared, hydrated, and stained with hematoxylin and eosin. The slides were examined and photographed with an Olympus BX51 microscope (Olympus, Japan). Strips of colonic tissue (15–30 mg from segments most intensively affected by the inflammation) were cut out, immersed in liquid nitrogen, and kept at −70°C until the cytokine measurement.

### 2.3. Real-time RT-PCR quantification of cytokine mRNA expression

Total RNA was isolated from the mouse colon (about 15 mg of tissue for each sample) with TRIzol reagent (Invitrogen Life Technologies, Karlsruhe, Germany) according to the manufacturer's instructions. Total RNA preparations were then cleaned and DNase I was treated with RNeasy Micro Kit (Qiagen, Hilden, Germany) according to the manufacturer's protocol. In order to quantify total RNA extracted from each sample, optical density at 260 nm was measured. The integrity of the RNA was assessed by denaturing agarose gel electrophoresis.

The RNA (0.75 *μ*g from each sample) was reverse transcribed at 42°C for 1 hour in 30 *μ*L containing 300 units of Superscript II Rnase H^-^reverse transcriptase (Invitrogen Life Technologies), 7 *μ*M anchored oligo dT13VN, 50 mM Tris-HCl pH 8.3, 3 mM MgCl_2_, 75 mM KCl, 10 mM DTT, 500 *μ*M dNTPs (dATP, dTTP, dCTP, dGTP), 60 units RNase OUT (recombinant ribonuclease inhibitor, Invitrogen Life Technologies), and 0.75 *μ*g acetylated BSA. The reaction was terminated by heating at 95°C for 5 minutes. To check for the presence of genomic DNA contamination in the RNA preparations, reverse transcriptase negative control (no reverse transcriptase in the reaction) was carried out in parallel, using RNA pool prepared from aliquots of all RNA samples. The pool of colon RNA obtained from aliquots of all samples served as standard RNA. The relative standard curve was generated using Mx 3000P 2.0 software (Stratagene, La Jolla, Calif).

PCR reactions were carried out in a 20 *μ*L final volume in duplicates using SYBRGreen I as a fluorescent detection dye. The reactions contained 0.8 *μ*L of cDNA (corresponding to 20 ng of sample total RNA), one unit of platinum Taq DNA polymerase (Invitrogen Life Technologies), SYBRGreen I in final dilution of 1 : 25000 (Sigma-Aldrich, Munich, Germany), 30 nM ROX (passive reference dye for correction of non-PCR-related fluctuations in fluorescence signal, Stratagene), 0.2 mM dNTPs (dATP, dTTP, dCTP, dGTP), 50 mM KCl, 10 mM Tris-HCl pH 8.3, 2.5 mM MgCl_2_(except for the *β*-actin reaction, where 1.5 mM MgCl_2_ was used), forward and reverse primers in final concentration of 0.25 *μ*M (for IL-1*β*, IL-12b, TNF*α*, IFN-*γ*, HPRT, and SDHA), or 0.5 *μ*M (for IL-6, IL-10, GM-CSF, and *β*-actin; see Table 2 for full gene names). Oligonucleotide primers used in the experiment were designed in our previous work [34], and their sequences are shown in Table 2. PCR amplification was performed in the real-time PCR system Mx 3000P (Stratagene). After an initial step at 95°C for 2 minutes (DNA denaturation and hot-start DNA polymerase activation), 40 cycles with the following thermocycling conditions were carried out: 94°C for 30 seconds, specific annealing temperature for 30 seconds, 72°C for 30 seconds, and specific temperature at which the fluorescence was acquired (“acquiring temperature”) for 30 seconds. Measurement of fluorescence at an elevated temperature (“acquiring temperature,” a few degrees of Celsius below the melting temperature of the specific PCR product) enables elimination of the fluorescence signal produced by incidental short nonspecific PCR products. Amplification specificity was then checked by generation of a melting curve using 41 cycles with temperature increments of 1°C (starting with 55°C) and a fluorescence measurement in each cycle. Specific annealing and acquiring temperatures are shown in Table 2.

To ensure the correctness of the quantification, we normalized cytokine expression to the expression of three housekeeping genes. Firstly, expression stability of several housekeeping genes was tested using geNorm software [35]. Subsequently, the normalization factor for each sample was calculated (by geNorm software) as the geometric means of the relative amounts of the three most stable housekeeping genes—HPRT, SDHA, and *β*-actin (see Table 2 for full gene names). Finally, the relative amount of cytokine mRNA in each sample was divided by the normalization factor of the sample.

### 2.4. Quantification of cytokine protein by ELISA

Colon tissue samples were homogenized in ice-cold PBS containing protease inhibitor cocktail for use with mammalian cell and tissue extracts (P8340, Sigma-Aldrich), and the homogenates were then centrifuged at 12000 xg at 4°C for 15 minutes. Total protein amounts in the tissue supernatants were determined using Bradford protein assay [36] with BSA employed as the standard. IL-1*β* and IL-6 amounts were determined using an ELISA kit, according to the manufacturer's recommendation (Pierce-Endogen, Rockford, Ill, USA).

### 2.5. Statistical analysis

The Kruskal-Wallis test and the Mann-Whitney U test were used for the estimation of macroscopic damage scores. The chi-square test was used to assess differences in mortality rate. The Student t test was used for the comparison of differences in body weight. The Kruskal-Wallis test was used for the comparison of differences in cytokine expression between groups and the Mann-Whitney U test was used to compare differences between the group of untreated colitic animals and other groups of animals. Values of p<.05 were considered as significant.

## 3. RESULTS

### 3.1. Body weight changes, mortality, and macroscopic damage scores

**Figure 1:**
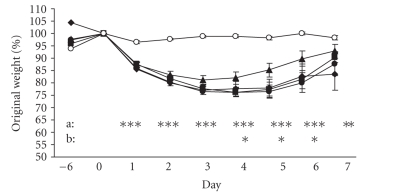
Body weight changes. Changes in body weight are expressed as a percentage of the original weight on day 0. Values are arithmetical means ± SEM. Statistical significance of the differences between the group of untreated colitic animals (TNBS group) and other groups of animals was assessed using the Student t test;*P≤.05, **P≤.01, ***P≤.001. A (*♦*): colitic animals fed with the combination of 0.4% thyme and 0.2% oregano oils; B (*▴*): colitic animals fed with the combination of 0.2% thyme and 0.1% oregano oils; C (*•*): colitic animals fed with the combination of 0.1% thyme and 0.05% oregano oils; D (*▪*): animals with TNBS-induced colitis; E (*∘*), control sham animals; a: statistical difference between the D and E groups; b: statistical difference between the D and B groups.

As shown in Figure 1, administration of TNBS caused a dramatic decrease in body weight (almost 20% after 3 days); body weight was recovered gradually from day 4 but not fully to the initial weight in day 7. Mice receiving 50% ethanol without TNBS (control sham group) showed only slight and transient loss of body weight. In mice of group B (colitic animals treated with 0.2% thyme and 0.1% oregano oils), body weight was recovered gradually from day 3 when it became higher than the body weight of untreated colitic animals (group D); on day 7, the body weight of animals in group B reached a level near to that of the control mice (sham group). The body weight of mice in group A (colitic animals treated with 0.4% thyme and 0.2% oregano oils) and group C (colitic animals treated with 0.1% thyme and 0.05% oregano oils) did not differ significantly from that of untreated colitic animals (group D).

**Figure 2:**
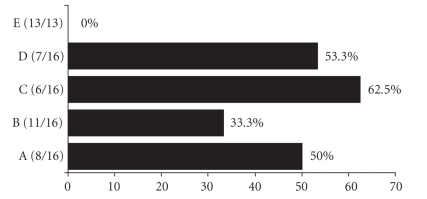
Mortality rate. Results are shown as the percentage of dead animals in each experimental group.Values in parentheses indicate the number of surviving animals compared with the total number of animals 7 days after induction of TNBS colitis. A, colitic animals fed with the combination of 0.4% thyme and 0.2% oregano oils; B, colitic animals fed with the combination of 0.2% thyme and 0.1% oregano oils; C, colitic animals fed with the combination of 0.1% thyme and 0.05% oregano oils; D, animals with TNBS-induced colitis; E, control sham animals.

The mortality rate of mice with TNBS-induced colitis (group D) was 53.3%, while that of the control sham group was 0% (see Figure 2). The mortality rate in group A (colitic animals treated with 0.4% thyme and 0.2% oregano oils) was 50% and in group C (colitic animals treated with 0.1% thyme and 0.05% oregano oils) was 62.5%, which are comparable to that found in group D. Treatment with the combination of 0.2% thyme and 0.1% oregano oils (group B) decreased the mortality rate to 33.3% (which was still not significantly different from that of mice with TNBS-induced colitis).

**Figure 3:**
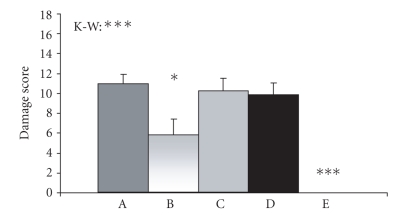
Colonic tissue damage. Macroscopic damage scores were assessed using Wallace's colon macroscopic scoring system (31). Values are arithmetical means + SEM, n=6−13. Statistical significance was assessed using the Kruskal-Wallis test (K-W, differences between all groups of animals) and Mann-Whitney test (difference between group D and other groups of animals). *P≤.05, ***P≤.001. A, colitic animals fed with the combination of 0.4% thyme and 0.2% oregano oils; B, colitic animals fed with the combination of 0.2% thyme and 0.1% oregano oils; C, colitic animals fed with the combination of 0.1% thyme and 0.05% oregano oils; D, animals with TNBS-induced colitis; E, control sham animals.

Macroscopic damage scores of mice in group D were significantly higher than those of mice in the control sham group. Treatment with the combination of 0.2% thyme and 0.1% oregano oils (group B) significantly lowered the macroscopic damage scores in comparison to untreated colitic animals (group D). Animals in groups A and C showed no significant changes in macroscopic damage scores compared with mice in group D (see Figure 3).

**Figure 4:**
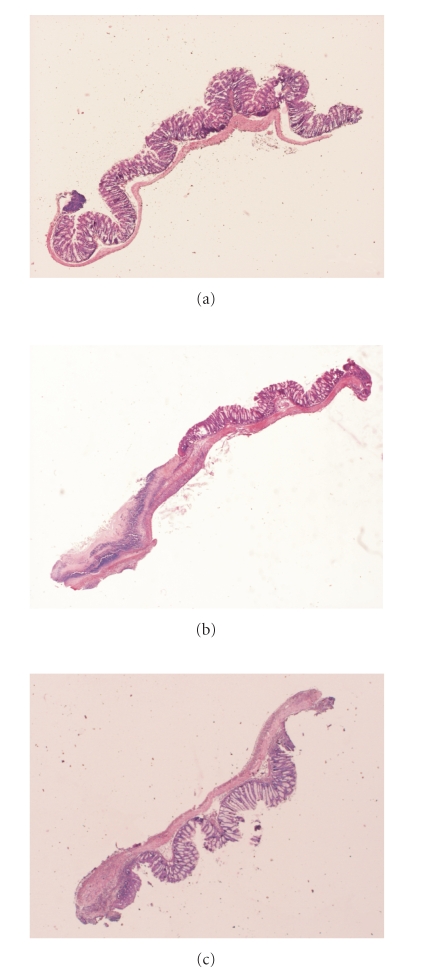
Histological appearance of mouse colonic tissue. Representative samples of colon after hematoxylin and eosin staining are shown. (a) Sham group; (E): no evident histological modification. (b) Mice with TNBS-induced colitis; (D) a wide range of histopathological changes including necrosis of epithelium, destruction of glands, and infiltration of inflammatory cells in the mucosa and submucosa (up to 50% of colon section). (c) Mice treated with 0.2% thyme and 0.1% oregano oils; (B) intermediary histopathological changes (up to 30% of colon section).

Representative samples of colon after hematoxylin and eosin stainings are shown in Figure 4. There is no evident histological modification in sham mice (E). In mice with TNBS-induced colitis (D), there is a wide range of histopathological changes including necrosis of epithelium, destruction of glands, and infiltration of inflammatory cells in the mucosa and submucosa (up to 50% of colon section). The samples from mice treated with 0.2% thyme and 0.1% oregano oils (B) show intermediary histopathological changes (up to 30% of colon section).

### 3.2. Expression of cytokine mRNA

**Figure 5:**
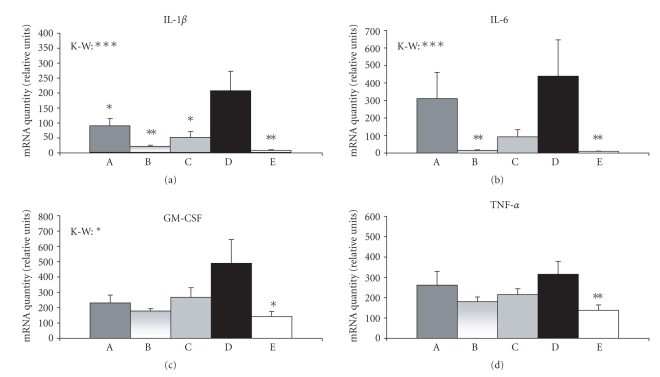
Cytokine mRNA expression. Relative amounts of a cytokine mRNA in each sample were divided by the normalization factor (geometric means of HPRT, SDHA and, *β*-actin amount) of the sample. Values are arithmetical means + SEM, n=6−8. Statistical significance was assessed using the Kruskal-Wallis test (K-W, differences between all groups of animals) and Mann-Whitney test (difference between group D and other groups of animals); *P≤.05, **P≤.01, ***P≤.001. A, colitic animals fed with the combination of 0.4% thyme and 0.2% oregano oils; B, colitic animals fed with the combination of 0.2% thyme and 0.1% oregano oils; C, colitic animals fed with the combination of 0.1% thyme and 0.05% oregano oils; D, animals with TNBS-induced colitis; E, control sham animals.

Relative amounts of IL-1*β*, IL-6, GM-CSF, and TNF*α* mRNAs (for full gene names, see Table 2) were significantly higher in animals with TNBS-induced colitis (group D) than in the control sham-treated animals (see Figure 5). Treatment of the colitic animals with the combination of thyme and oregano oils significantly lowered the amount of IL-1*β* mRNA, using all three tested doses of the aromatic oils (see Figure 5). The amount of IL-6 mRNA in group B (colitic animals treated with 0.2% thyme and 0.1% oregano oils) was significantly lower than that in the group D, whereas the decrease of IL-6 mRNA level in groups A and C (colitic animals treated with 0.4% thyme and 0.2% oregano oils or with 0.1% thyme and 0.05% oregano oils) was not statistically significant (see Figure 5). A similar effect was found for GM-CSF and TNF*α* but the difference between group B and group D did not reach statistical significance (p=.064 and p=.053, resp.; see Figure 5). We found no significant changes in mRNA levels of two other cytokines (IL-10 and IFN*γ*, data were not shown).

### 3.3. Expression of IL-1*β* and IL-6 proteins

**Figure 6:**
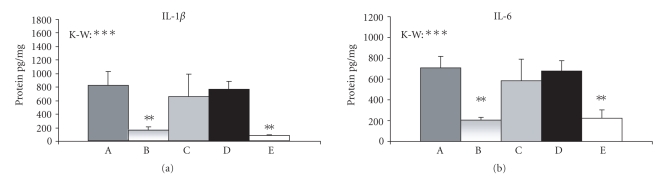
IL-1*β* and IL-6 protein expression. Values are arithmetical means + SEM, n=6−8. Statistical significance was assessed using the Kruskal-Wallis test (K-W, differences between all groups of animals) and Mann-Whitney test (difference between group D and other groups of animals); **P≤.01, ***P≤.001. A, colitic animals fed with the combination of 0.4% thyme and 0.2% oregano oils; B, colitic animals fed with the combination of 0.2% thyme and 0.1% oregano oils; C, colitic animals fed with the combination of 0.1% thyme and 0.05% oregano oils; D, animals with TNBS-induced colitis; E, control sham animals.

As shown in Figure 6, the amounts of IL-1*β* and IL-6 proteins were significantly higher in the animals with TNBS-induced colitis (group D) than in the control sham group. The concentrations of these proteins were significantly reduced in group B (colitic animals treated with 0.2% thyme and 0.1% oregano oils) compared to group D. The levels of IL-1*β* and IL-6 proteins in groups A and C (colitic animals treated with 0.4% thyme and 0.2% oregano oils or with 0.1% thyme and 0.05% oregano oils) did not differ significantly from those found in group D (see Figure 6).

## 4. DISCUSSION

Various aromatic plants and their products have been reported to have health benefit properties. In this study, we examined whether dietary supplementation with a combination of thyme essential oil and oregano essential oil could have a protective effect in intestinal inflammation. We applied three doses of thyme and oregano essential oil combination (0.4% thyme and 0.2% oregano oils, 0.2% thyme and 0.1% oregano oils, 0.1% thyme and 0.05% oregano oils) to mice with TNBS-induced colitis, and found that administration of the medium dose decreased the mortality rate from 53% to 33%, significantly accelerated the body weight gain recovery, and significantly reduced the macroscopic damage of the colonic tissue. In animals fed with the two other doses of the essential oils, we did not find a significant improvement in the mortality rate, body weight gain recovery, or colonic tissue damage.

Examining the expression of cytokines, we found significantly elevated mRNA levels of proinflammatory cytokines IL-1*β*, IL-6, GM-CSF, and TNF*α* in mice with TNBS-induced colitis. This finding is in agreement with other studies showing increased expression of proinflammatory cytokines in mouse experimental models of colitis [4–7]. Treatment of the colitic animals with the medium dose of aromatic oils (0.2% thyme and 0.1% oregano oils) decreased the mRNA amounts of IL-1*β*, IL-6, GM-CSF, and TNF*α* (in GM-CSF and TNF*α*, the difference did not reach statistical significance). Other two doses (0.4% thyme and 0.2% oregano oils; 0.1% thyme and 0.05% oregano oils) were less effective and the difference in cytokine mRNA level between animals treated with these doses of the aromatic oils and untreated colitic animals was significant only in the case of IL-1*β*.

The most sensitive indicators of the effectiveness of the thyme and oregano oil treatment were mRNA levels of proinflammatory cytokines IL-1*β* and IL-6 in our experiment. Similarly, Kwon et al. [7] demonstrated that plant flavonoid rutin administered in diet to mice with dextran sulfate sodium (DSS)-induced colitis significantly suppressed mRNA levels of IL-1*β* and IL-6, whereas the effect on mRNA level of GM-CSF was less marked. Increase of IL-1*β* and IL-6 mRNA levels in inflamed intestinal tissue has been well documented [12–14], and there are data indicating that IL-1*β* can stimulate production of IL-6 in intestinal epithelial cells [37, 38]. Reported data about the expression of TNF*α* are somewhat contradictory, showing either no change or an increase of TNF*α* levels in intestinal inflammation [10]. Sugimoto et al. [4] showed that elevated levels of TNF*α* mRNA in mice with TNBS-induced colitis can be suppressed by curcumin treatment, but Kwon et al. [7] found no significant changes in TNF*α* mRNA levels in mice with DSS-induced colitis (treated or untreated with plant flavonoid rutin).

Examination of IL-1*β* and IL-6 protein expression in our experiment confirmed the best efficacy of the medium dose of thyme and oregano oil combination in the suppression of colitis. The cytokine protein level in animals treated with the medium dose, but not in animals treated with the other two doses, was significantly lower than in the untreated colitic animals. The low effectiveness in attenuating the colitis found after administration of the highest dose of thyme and oregano oil combination could be connected with possible cytotoxic effects of higher concentrations of these oils. In our preliminary experiments with intact mice, we found negative effects of higher concentrations of thyme essential oil (1%) and oregano essential oil (0.5%) on body weight and food intake. Moreover, the cytotoxic effect of higher concentrations of these oils has been demonstrated in intestinal cells and lymphocytes [39, 40]. Thus, the lack of the dose dependency found in this study could be due to a combination of positive and negative effects of the highest concentration of thyme and oregano essential oil combination. On the other hand, too low doses of essential oils can be insufficient to reduce the intensity of inflammation. A similar phenomenon was found in the study examining the protective effects of curcumin on TNBS-induced colitis in mice, where the medium tested dose was more effective at improving body weight gain than the lower or higher doses of curcumin. To explain the lack of the dose dependency, authors speculated about curcumin toxicity or about the influence on the food intake [4]. Thus, the use of optimal doses is essential for good efficacy of essential oils (or their components) in attenuating inflammation. Further experiments are needed to establish the most efficient concentration of thyme and oregano essential oil combination.

In our preliminary experiments (using similar experimental conditions as in the present study), we administered thyme (0.5%, 0.25%, 0.12%) or oregano (0.4, 0.2%, 0.1%) essential oil alone in diet to mice with TNBS-induced colitis and we found no significant positive effect. Furthermore, we observed negative effects on body weight and food intake with oregano at 0.4% concentration. However, we observed a better recovery of body weight after treatment with a combination of these essential oils in preliminary tests. The results of the present study confirm the positive effects of the combined administration of appropriate concentrations of thyme and oregano oils on TNBS-induced colitis. It is known that oregano essential oil possesses strong antimicrobial activity, which is ascribed to carvacrol, the main component of this oil [41]. Thymol, one of the major compounds of thyme essential oil, has anti-inflammatory activity [21]. Thus, it seems that major components of these oils can complement one another, having synergic effects on inflammation so that their combination could exhibit positive preventive or therapeutic effects. A similar phenomenon was found in another study where the effect of peppermint oil and caraway oil on postinflammatory visceral hyperalgesia was examined (using a rat model of TNBS-induced colitis). Neither peppermint nor caraway oil administered individually had a significant effect on postinflammatory visceral hyperalgesia, but combined treatment with these essential oils significantly reduced the visceromotor response [42].

Mechanisms mediating the suppressive effects of thyme and oregano oils on colitis are unclear, and we can only speculate that there are several potential manners of action. One possibility could be the influence of the essential oils on nuclear factor *κ*B (NF-*κ*B), a pleiotropic transcription factor which can activate expression of genes involved in immune and inflammation responses such as proinflammatory cytokines [43]. Several studies have demonstrated an inhibitory effect of various plant extracts or their components on NF-*κ*B activation. Suppression of the NF-*κ*B inhibitory protein (I*κ*B) degradation in colonic epithelial cells and macrophages was demonstrated after the administration of curcumin and zerumbone [4, 44, 45]. Reduced activation of NF-*κ*B was also shown after treatment with black tea polyphenol theaflavin in RAW 264.7 cells [46]. On the other hand, IL-1*β* (such as other proinflammatory cytokines) is a potent inducer of NF-*κ*B [43] and it has been shown that extract of *Thymus pulegioides* can inhibit activation of NF-*κ*B by IL-1*β* in human hepatoma cells [47].

In conclusion, the present data indicate that dietary administration of a combination of thyme and oregano essential oils in appropriate concentrations can reduce the production of proinflammatory cytokines and attenuate the degree of colonic tissue injury, and thereby ameliorate TNBS-induced colitis in mice. We suggest that the combination of thyme and oregano essential oils has potential value as an additional or supporting treatment in gastrointestinal inflammations. Our results indicate that some essential oils could have positive effects on TNBS-induced colitis but seemingly in rather narrow range of concentrations, thus limiting their therapeutic/preventive potential.

## Figures and Tables

**Table 1:** Experimental groups of animals.

	Treatment
Group A	Mice with TNBS-induced colitis fed with 0.4%—4000 ppm (wt/wt)—thyme oil + 0.2%—2000 ppm (wt/wt)—oregano oil
Group B	Mice with TNBS-induced colitis fed with 0.2%—2000 ppm (wt/wt)—thyme oil + 0.1%—1000 ppm (wt/wt)—oregano oil
Group C	Mice with TNBS-induced colitis fed with 0.1%—1000 ppm (wt/wt)—thyme oil + 0.05%—500 ppm (wt/wt)—oregano oil
Group D	Mice with TNBS-induced colitis
Group E	Sham-treated mice

**Table 2:** Oligonucleotide primers for real-time PCR.

Gene name${}^{\text{a}}$	Primer sequence (5′-3′)${}^{\text{b}}$	Ta${}^{\text{c}}$(°C)	Tacq${}^{\text{d}}$(°C)
IL-1*β*	FP: AAGTGATATTCTCCATGAGCTTTGT	64	82
	RP: TTCTTCTTTGGGTATTGCTTGG		
IL-6	FP: TGGGAAATCGTGGAAATGAG	66	80
	RP: CTCTGAAGGACTCTGGCTTTG		
IL-10	FP: CAACATACTGCTAACCGACTCCT	66	82
	RP: TGAGGGTCTTCAGCTTCTCAC		
TNF*α*	FP: CGTCGTAGCAAACCACCAAG	64	80
	RP: TTGAAGAGAACCTGGGAGTAGACA		
GM-CSF	FP: GCAATTTCACCAAACTCAAGG	64	82
	RP: CTCATTACGCAGGCACAAAAG		
IFN*γ*	FP: ATCAGGCCATCAGCAACAAC	62	82
	RP: ATCAGCAGCGACTCCTTTTC		
*β*-actin	FP: AAATCGTGCGTGACATCAAAG	70	82
	RP: AAGAAGGAAGGCTGGAAAAGAG		
HPRT	FP: TGGATACAGGCCAGACTTTGTT	68	80
	RP: ACTTGCGCTCATCTTAGGCTTT		
SDHA	FP: CATGCCAGGGAAGATTACAAAG	65	80
	RP: AGTAGGAGCGGATAGCAGGAG		

${}^{\text{a}}$IL, interleukin; TNF*α*, tumor necrosis factor alpha; GM-CSF, granulocyte-macrophage colony stimulating factor; IFN*γ*, interferon gamma; *β*-actin, beta actin; HPRT, hypoxanthine guanine phosphoribosyl transferase 1; SDHA, succinate dehydrogenase complex subunit A

${}^{\text{b}}$FP, forward primer; RP, reverse primer

${}^{\text{c}}$Ta, annealing temperatures used for thermal cycling

${}^{\text{d}}$Tacq, temperature at which the fluorescence signal was acquired (“acquiring temperature”)
